# How Accurate Is Oral Implant Installation Using Surgical Guides Printed from a Degradable and Steam-Sterilized Biopolymer?

**DOI:** 10.3390/jcm9082322

**Published:** 2020-07-22

**Authors:** Stefano Pieralli, Benedikt Christopher Spies, Valentin Hromadnik, Robert Nicic, Florian Beuer, Christian Wesemann

**Affiliations:** 1 Department of Prosthetic Dentistry, Center for Dental Medicine, Medical Center—University of Freiburg, Faculty of Medicine—University of Freiburg, 79106 Freiburg, Germany; stefano.pieralli@uniklinik-freiburg.de; 2 Department of Prosthodontics, Humboldt-Universität zu Berlin, and Berlin Institute of Health, Charité—Universitätsmedizin Berlin, corporate member of Freie Universität Berlin, Geriatric Dentistry and Craniomandibular Disorders, 14197 Berlin, Germany; valentin.hromadnik@charite.de (V.H.); robert.nicic@charite.de (R.N.); florian.beuer@charite.de (F.B.); christian.wesemann@charite.de (C.W.); 3 Department of Orthodontics, Humboldt-Universität zu Berlin, and Berlin Institute of Health, Charité—Universitätsmedizin Berlin, corporate member of Freie Universität Berlin, Dentofacial Orthopedics and Pedodontics, Aßmannshauser Str. 4-6, 14197 Berlin, Germany

**Keywords:** accuracy, additive manufacturing, biodegradability, computer-aided design, dental implants, fused deposition modelling, medical device, lignin, surgical guides

## Abstract

3D printed surgical guides are used for prosthetically-driven oral implant placement. When manufacturing these guides, information regarding suitable printing techniques and materials as well as the necessity for additional, non-printed stock parts such as metal sleeves is scarce. The aim of the investigation was to determine the accuracy of a surgical workflow for oral implant placement using guides manufactured by means of fused deposition modeling (FDM) from a biodegradable and sterilizable biopolymer filament. Furthermore, the potential benefit of metal sleeve inserts should be assessed. A surgical guide was designed for the installation of two implants in the region of the second premolar (SP) and second molar (SM) in a mandibular typodont model. For two additive manufacturing techniques (stereolithography [SLA]: reference group, FDM: observational group) n = 10 surgical guides, with (S) and without (NS) metal sleeves, were used. This resulted in 4 groups of 10 samples each (SLA-S/NS, FDM-S/NS). Target and real implant positions were superimposed and compared using a dedicated software. Sagittal, transversal, and vertical discrepancies at the level of the implant shoulder, apex and regarding the main axis were determined. MANOVA with posthoc Tukey tests were performed for statistical analyses. Placed implants showed sagittal and transversal discrepancies of <1 mm, vertical discrepancies of <0.6 mm, and axial deviations of ≤3°. In the vertical dimension, no differences between the four groups were measured (*p* ≤ 0.054). In the sagittal dimension, SLA groups showed decreased deviations in the implant shoulder region compared to FDM (*p* ≤ 0.033), whereas no differences in the transversal dimension between the groups were measured (*p* ≤ 0.054). The use of metal sleeves did not affect axial, vertical, and sagittal accuracy, but resulted in increased transversal deviations (*p* = 0.001). Regarding accuracy, biopolymer-based surgical guides manufactured by means of FDM present similar accuracy than SLA. Cytotoxicity tests are necessary to confirm their biocompatibility in the oral environment.

## 1. Introduction

Implant-supported single crowns (SCs) and multi-unit fixed dental prostheses (FDPs) present a predictable treatment option for patients with missing or hopeless natural teeth [1,2]. However, biological and technical complications can be associated with malpositioned implants or poorly designed superstructures [3,4,5]. Backward planning, as available in digital workflows, can be considered an effective tool to avoid implant malposition [6,7].

An implant planning software allows to import acquired radiographical and surface data and to visualize the final outcome, thereby facilitating to define the ideal implant site according to a virtual set-up and the anatomical characteristics of the patient [8,9]. Based on the virtually planned implant position, a surgical drilling guide can be manufactured, e.g., by 3D printing [10,11] and adapted to the clinical workflow without a typical learning curve [12]. Printing technology based on computer aided design and manufacturing (CAD-CAM) allows to create objects by adding consecutive layers [13]. Surgical guides made from resin-based materials using the stereolithography (SLA) or digital light processing (DLP) technique are mostly used in daily clinical routine [14] also for skeletal anchorage purposes [15]. Using SLA for printing, multiple layers of fluid resin are successively photopolymerized by selective ultraviolet (UV) laser radiation (λ = 350–450 nm) allowing the creation of highly accurate objects [16] with fine grained complex geometries [17]. However, increased manufacturing costs and time-consuming post processing procedures are necessary for manufacturing SLA printed surgical guides, and potential debris by means of detached resin particles are likely to affect the surgical site. Additional non-printable stock articles such as metal guidance in the form of sleeves or drill handles are frequently used to avoid wear and debris of the guide and incorrect drilling paths [18].

To date, multiple 3D printing technologies available on the market are suitable for additive manufacturing of dental devices such as surgical guides [19], among these fused deposition modelling (FDM) represents a straightforward and cost-effective method [20]. This technique allows extrusion of melted thermoplastic filaments through a mobile nozzle with subsequent deposition on a hot plate according to the planned CAD dataset, requiring a minimal postprocessing sequence [21,22]. Despite the favorable characteristics of this technique, accuracy of the final object is still a topic of controversial discussion and seems to be highly dependent of the printing process and the raw material used [23]. 

Among other filaments, FDM technology allows for additive manufacturing of biopolymers such as lignin and polylactides (PLA), a biodegradable filament used for tissue engineering purposes in various fields of regenerative medicine and dentistry [24,25,26]. Despite environmental advantage in terms of biodegradability, recent studies showed improvements of the mechanical properties by changing the layups of the fibers of subsequent layers [20]. Since FDM printed surgical guides made of lignin and PLA might represent a cost-effective, sterilizable, and environmental-friendly alternative to surgical guides made of SLA printed resins, this study aimed to compare SLA and FDM printed guides in terms of the accuracy when used for implant positioning.

The purpose of this investigation was to measure the accuracy of a guided workflow for implant placement by comparing the discrepancies between the virtually planned (target) and finally realized position of oral implants at the level of the apex, shoulder, and the implant axis. The influence of the manufacturing technology (SLA and FDM) and a potential benefit of inserted metal sleeves were evaluated on the basis of two implants installed to support a three-unit FDP in a mandibular model. 

The null hypothesis assumed no significant horizontal or vertical discrepancies between target and realized implant position, irrespective of the 3D printing method and the use of metal guidance at both implant positions. 

## 2. Experimental Section

### 2.1. Reference Model

A typodont model (IMP1001-UL-SP-DM, Nissin, Kyoto, Japan) revealing a removable edentulous alveolar ridge located in the posterior part of the lower left quadrant was used to install two two-piece titanium oral implants in the region of the second premolar and molar (SP and SM), respectively. To allow multiple implant placement, the removable alveolar ridge of the model was digitized using an intraoral scanner (IOS) (TRIOS 3, 3shape, Copenhagen, Denmark), adjusted in an inspection-software (Fusion 360, Autodesk, San Rafael, CA, USA) and 3D printed from a ABS filament (DuraPro ABS black, Exdrudr, Lauterach, Austria) by means of FDM, with an 1.5 mm outermost layer printed in compact form (100%) and an infill of 75%. This aimed to mimic cortical and spongiose alveolar bone. 

### 2.2. Digital Implant Planning

**Figure 1:**
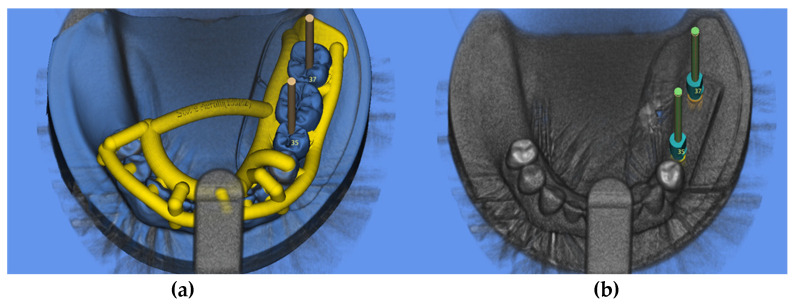
Digital implant planning: (**a**) design of the surgical guide, (**b**) virtual application of scan bodies (target position).

To simulate a clinical workflow, a cone-beam computer tomography (CBCT) (Veraviewepocs 3-D R100, J. Morita Corp., Osaka, Japan) of the reference model was performed. The mandible was removed from the occludator by a medical technical assistant prior to radiography to avoid metal-induced artifacts (field of view: 8 cm × 8 cm, circulation time: 9.4 s, tube voltage: 80 kV and current intensity: 1 mA). The corresponding virtual dataset (Digital Imaging and Communications in Medicine, DICOM) was subsequently imported in an implant planning software (SMOP, Swissmeda AG, Baar, Switzerland) and superimposed with the surface data (standard tessellation language, STL) obtained with an IOS (TRIOS 3, 3shape) of the reference model by a professional in the field of digital design. To avoid distortions, a standardized scan path, under ceiling lighting was followed during the acquisition of the STL data. Thereafter, a three-unit FDP (ranging from the SP to the SM) was designed by a master dental technician (R.N.) using a CAD software (Dental Designer, 3shape) and imported into the implant-planning software (Figure 1). 

Two two-piece titanium implants were virtually positioned and parallelized in the afore mentioned regions to allow for transocclusal screw-retention of the FDP. Thereafter, implant positions (referred to by virtual scan bodies) were exported as STL file (“target position”). Finally, a surgical template for guided implant installation was designed (J.B.). For sleeveless (N.S.) insertion, the diameter of the drilling holes of the surgical guides was digitally adapted to an inner diameter of 4.6 mm in order to match the inner diameter of the used titanium sleeves (S) (Guide System Guiding sleeve, REF.: J3734.3803, Camlog). 

### 2.3. 3D Printing of Surgical Guides

For each of the four groups (FDM-S, FDM-NS, SLA-S, SLA-NS) 10 surgical guides were additively manufactured and steam sterilized in an autoclave (15 min, 121 °C) (Webeco, Serie EC, Selmsdorf, Germany). Two different 3D printing technologies were evaluated in this investigation. SLA (Form 2, Dental SG Resin, Formlabs, Boston, MA, USA) was used to manufacture surgical guides made of medical class 1 resin (Dental SG Resin, Formlabs) certified for dental use (EN-ISO 10993-1:2009/AC:2010, USP Class VI) by printing 0.05 mm thin layers. The average printing time of each sample was 50 min. This allowed simultaneous printing of multiple units followed by a post-processing sequence consisting of a 5 min rinsing in alcohol (99% isopropanol), air-drying, and 30 min light curing (λ = 405 nm) at 60 °C. Finally, printing supports were removed, and smooth surface finishing was performed. The FDM printed surgical guides (Prusa i3 MK3, Prague, Czech Rep.) were manufactured using an experimental biofilament based on lignin with a layer thickness of 0.06 mm, a printing temperature of 220 °C and a print bed temperature of 60 °C. The average printing time was 60 min. Apart of removing the support-structures and polishing, no further post-processing was needed for FDM printed surgical guides. Finally, the guides were steam-sterilized as the SLA printed guides mentioned afore. 

### 2.4. Surgical Protocol

The surgical guides were used for the insertion of 80 two-piece regular platform tapered titanium implants (3.8 × 9 mm; CAMLOG^®^ SCREW-LINE Promote plus, REF.: K1052.3809; Camlog, Basel, Switzerland) in the removable part of the mandible model following a standardized drilling protocol. In the first instance, a pilot drill with a diameter of 2 mm (Guide System Pilot drill set, REF.: J5063.4309; Camlog) (800 rounds per minute, RPM) provided with calibrated depth control was used. Subsequent, two successive yellow-color coded drills (Guide System Surgery-set SCREW-LINE, REF.: J5065.3809; Camlog) designated for the installation of 3.8 mm wide implants of this type were provided with metal sleeves and used to progressively enlarge the implant site (500 RPM). Finally, a cortical bone drill expanded the implant site (Guide System Form drill SCREW-LINE, Cortical bone, REF.: J5068.3809; Camlog) (500 RPM) and manual tapping (15 RPM) was performed. All implants were inserted by hand (<15 RPM) and provided with scan bodies (CAMLOG^®^ Scanbodies, REF.: K2610.3810, Camlog), made of polyetheretherketon (PEEK). To simulate a clinical setting, drilling and implant insertion procedures were performed using a dental simulator manikin phantom head by one single operator (V.H.).

### 2.5. Data Acquisition 

**Figure 2:**
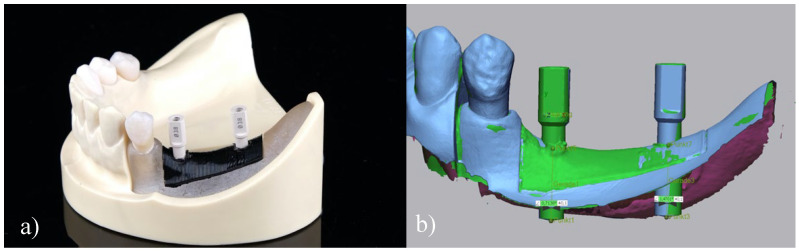
Digitization of the scan bodies: (**a**) Scan bodies in situ (implant final position), (**b**) Superimposition of planned and final position.

The digitization process of the scan bodies was conducted by means of a 3D LED scanner (D2000, 3shape) showing a precision of 5 μm/8 μm (ISO 12836/implant). The resulting STL files were subsequently imported in an inspection software (Geomagic Control X, 3-D Systems; Rock Hill, CA, USA) and aligned with the target implant position. For this purpose, the actual 3D position of the scan bodies was used as reference and superimposed to the target position by performing an initial 7-point alignment followed by a best-fit superimposition according to Gauss (Figure 2). Thereafter, sagittal, transversal and vertical discrepancies were measured at both the implant apex and implant shoulder level using a linear 2-point measurement. Similarly, deviations of the main axis were assessed. 

### 2.6. Statistical Analysis

For the statistical analysis, data were tested for normal distribution (Kolmogorow–Smirnow test) and variance homogeneity (Levene test). Afterwards multivariate analyses of variance (MANOVA) with post hoc Tukey tests were calculated for the independent variables 3D printing technology (SLA, FDM) and use of sleeve (S, NS) at both implant positions (SP and SM). The statistical analysis was calculated with a statistical software program (IBM SPSS Statistics, v22.0, IBM Corp). The significance level was set at α = 0.05. 

## 3. Results

**Figure 3:**
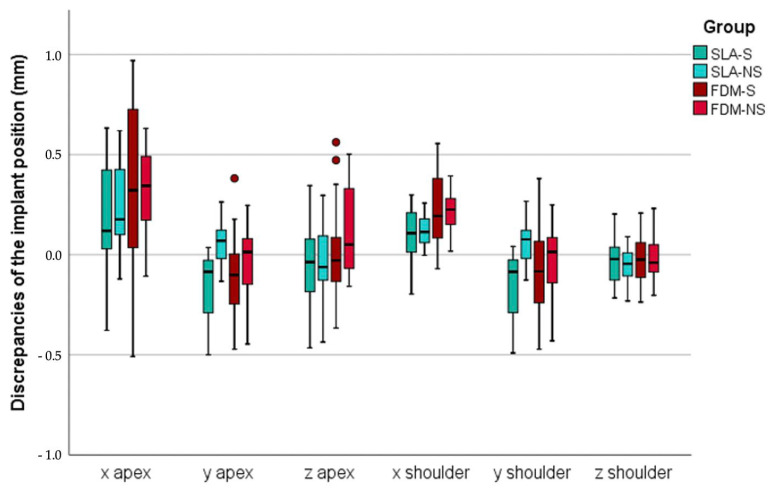
Boxplots of minimum, maximum, interquartile range, median, and outliers for discrepancies of the implant position at implant apex and shoulder level. SLA: stereolithography, FDM: fused deposition modeling, S: metal sleeve, NS: no metal sleeve, x: sagittal discrepancies, y: transversal discrepancies, z: vertical discrepancies.

**Figure 4:**
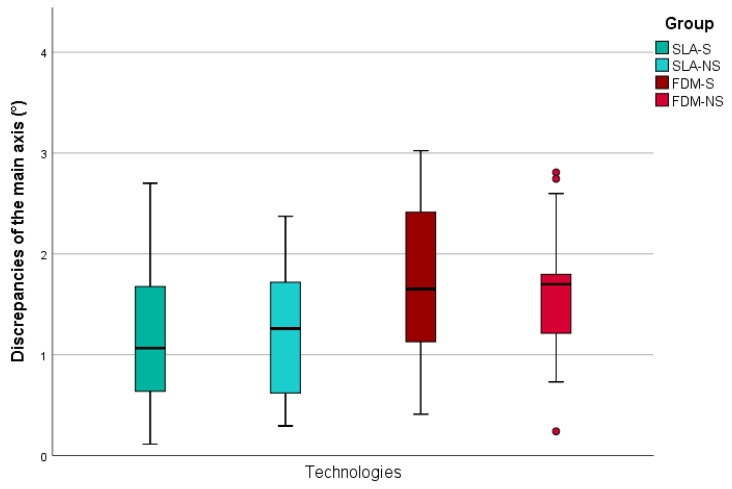
Boxplots of minimum, maximum, interquartile range, median, and outliers for discrepancies of the implant main axis. SLA: stereolithography, FDM: fused deposition modeling, S: metal sleeve, NS: no metal sleeve, x: sagittal discrepancies, y: transversal discrepancies, z: vertical discrepancies.

A total of 80 implants were inserted by using four different groups (n = 10) of surgical guides (SLA-S, SLA-NS, FDM-S, FDM-NS). Compared to target, all installed implants showed maximum vertical (z) deviations of <0.6 mm and horizontal (x, y) deviations of <1.0 mm (Figure 3). A maximum angular discrepancy referring to the main axis of 3.02° was calculated (Figure 4). Mean and descriptive statistics can be found in Table 1. MANOVA revealed significant transversal (y) differences at the apex (*p* = 0.001), sagittal (x) and transversal (y) differences at the shoulder level (*p* = 0.001) and axial differences (*p* = 0.033) between the groups (Table 2).

### 3.1. Sagittal Discrepancies (X)

No significant deviations were measured at the apical level of the implants between the groups (*p* = 0.05). However, at the implant shoulder, significant sagittal differences were found in the SM region (*p* = 0.001). Both SLA groups showed decreased discrepancies compared with FDM (*p* ≤ 0.033). The use of a metal sleeve did not affect the accuracy of implant insertion for both SLA (*p* = 0.992) or FDM (*p* = 0.482).

### 3.2. Transversal Discrepancies (Y)

Mean transverse discrepancies at the apical level were found to be 0.4 ± 0.2 mm with deviations ranging from 0.1 to 0.9 mm. Apically, both printing technologies showed comparable results, but sleeveless insertion revealed lower deviations in the SP region for both SLA (*p* = 0.001) and FDM (*p* = 0.201). Similarly, minor differences between the printing technologies were found at the implant shoulder, but again sleeveless insertion increased the accuracy for both SLA (*p* = 0.001) and FDM (*p* = 0.001) in the SP region. 

### 3.3. Vertical Deviations (z)

Irrespective of the group, the mean vertical deviation resulted in 0.2 ± 0.1 mm at the apex level and 0.1 ± 0.1 mm at the shoulder level. Overall maximum vertical discrepancies ranged between 0.1 and 0.6 mm at the apex level, and 0.1 and 0.2 mm at the shoulder level. MONAVA showed neither apical (*p* ≥ 0.054) nor at implant shoulder level (*p* ≥ 0.352) differences between the groups in both implant regions. 

### 3.4. Main Axis Deviations

No significant axial differences between the groups were found in the SP region (*p* = 0.409) with mean deviations ranging between 1.3 ± 0.9° (SLA-S) and 1.9 ± 0.8° (FDM-S). Likewise, comparable results between the groups were measured in the area of SM. Only the group with the smallest mean deviations of 0.8 ± 0.5 (SLA-NS) differed significantly from the largest deviations of 1.7 ± 0.9 (FDM-S) (*p* = 0.020).

## 4. Discussion

The aim of this investigation was to assess the accuracy of guided implant positioning by using a simplified and more economic additive CAM method, namely FDM, and compare it to SLA. A total of 80 two-piece titanium implants were positioned and digitized. Significant differences between final and planned implant position resulted comparing FDM and SLA, and the null hypothesis was partially rejected. Regardless of the study group, maximum vertical deviations of <0.6 mm, horizontal deviation of <1 mm and axial deviation of ≤3.02° were measured.

In this study implants were placed in vitro in a typodont model, which does not fully reflect the clinical situation. Especially differences between natural bone and resin blocks as implant site can affect implant installation. The rational was therefore to create a bi-layered alveolar bone architecture by creating blocks with 1.5 mm external layers printed in compact form with 100% infill (cortical bone) and an internal part with 75% infill, simulating a porous scaffold (spongiosa). As proof of concept, three different internal densities of infill (50%, 75%, and 100%) were used to perform test implant placements at study conceptualization by five experienced clinicians. Eligibility criteria were a resistance to drilling comparable to the posterior mandible (2/3 bone quality) [27] and implant insertion torque < 30 Ncm (recommended for this implant system). Blocks with 75% intern infill fulfilled both above mentioned criteria and were adapted.

The final implant position was assessed by digitization of the scan body and superimposing the planed and final implant position. This approach has already been described in vitro [28] and in vivo [29,30]. In a recent RCT, a postoperative CBCT, with the same field of view of the diagnostic one, was conducted to evaluate the accuracy of guided implant positioning as an alternative method [31] showing comparable results to digitization of scan bodies [32]. 

Implant planning software are CAD systems which virtually recreate the intraoral circumstances for planification purposes. Comparable to other implant software, the one used in this investigation (SMOP) enables to match a 3D radiographic dataset with digitized surface geometries [33]. X-ray data for 3D virtual planning purposes are produced by CBCT and saved as voxel based volumetric DICOM files, whereas the intraoral scans and the wax up/set up of the final restoration are saved as surface polygons in STL format. According to the results of a prospective controlled clinical investigation by Schnutenhaus et al. manual alignment between CBCT and model scan data performed using afore mentioned software can lead to mean deviations of 0.2 mm [34]. However, matching CBCT and surface scans and 3D printing surgical guides using SMOP lead to a more accurate implant positioning compared to surgical guides which use physical positioners to transfer the coordinates of the planned drilling slots [35]. Since dental surfaces are considered ideal for matching purposes, a sufficient number of teeth with few restoration-induced artifacts are needed for manual matching procedures and avoid the use of an x-ray template as reference [8]. 

Producing surgical guides by FDM was found to be more cost-effective and less time consuming than using SLA. Considering the FDM printing equipment used in this study, a sleeveless surgical guide can be produced with material costs 0.5–1 € compared to 10–20 € if produced by means of SLA. Furthermore, 50 min printing time and additional 35 min post-processing (5 min washing + 30 min light curing) were needed to manufacture an SLA printed surgical guide. An FDM printed surgical guide was fabricated in 65 min (60 min printing time + 5 min post processing).

For this investigation a typodont model of a mandible with unilateral shortened dental arch beginning from the SP was used to plan a screw retained three-unit implant supported FDP (SP–SM). Implants in both locations showed comparable deviations. In a clinical study no difference between implants placed using free ending surgical guides in a shortened dental arch compared with mesial and distal tooth-supported surgical guides in an interrupted arch was measured [36]. This is consistent with the study results of Schnutenhaus et al. evaluating the accuracy of implant position as a function of the remaining teeth [37]. The wide basal configuration designed for mucosal-supported areas seems to balance the soft tissue resilience (snow-shoe principle). 

Surgical guides with (S) and without sleeves (NS) showed an equal drill-slot gap (intrinsic error) [38] and the advantage of using metal guidance for implant placement was questioned. In this investigation the use of metal sleeves (S) did not result in more accurate implant positioning compared to NS for both the printing methods. The avoidance of metal sleeves reduced costs (one package containing two-sleeves costs 33 €), production time and error potentials (luting of the sleeve into the slot). Laederach et al. evaluated several systems provided with metal guidance in terms of accuracy of final implant position and could show significant deviations comparing centric and eccentric drilling procedures [39]. On one side, the use of metal guidance does not seem to prevent the clinician from eccentric drilling paths. On the other hand, horizontal drifting of the rotating drill from the metallic slot leads to overheating of the drill and metallic debris, both potentially detrimental for the implant site [40,41]. Metallic particles in the surrounding tissue of the implant are discussed as a potential cofactor for periimplantitis [42]. A sleeveless system that allows for hand-piece guidance rather than drill guidance, thereby avoiding direct contact of rotating components with the surgical guide, is available on the market but information regarding its accuracy is still scarce [43]. In contrast, the approach of this study was to produce surgical guides from a printable and potentially biodegradable biopolymer as proof of concept. A biodegradable material that is suitable for additive manufacturing is PLA. However, pure PLA transforms into the glass phase during autoclave sterilization at temperatures of 121 °C and will potentially deform/lose dimensional accuracy. Likewise overheating due to contact with the drill could lead to deformation of the material. For this reason, a novel lignin-containing filament was investigated for the production of medical products. Lignin is a cross-linked phenolic polymer that occurs in the cell walls of plants and is thermally stable up to 165 °C [44]. 

Main limitation of the present investigation consisted in the in vitro design, which might have led to more accurate results compared to clinical reality. However, the experimental 3D printable biofilament is not yet allowed for clinical use. Potential bias might be related to the identical design of the surgical guides and the single operator positioning the implants. In addition, changing the manufacturing parameters (e.g., thickness of the layers, printing temperature) could have resulted in a different outcome. To the best knowledge of the authors, this was the first attempt to use the investigated biopolymer to fabricate cost effective, degradable, and sterizable surgical guides for accurate implant positioning by FDM.

In this study, the lignin drilling templates produced by FDM allowed a reduction of the production costs of 50% even if metallic sleeves are used. However, metal sleeves did not lead to a more accurate implant position. Production of FDM-NS surgical guides resulted the most cost-effective (0.5–1 € per unit) and SLA-S the most expensive (40–50 € per unit). Therefore, further research should address the cytotoxicity of the biofilament for medical use approval and the potential biodegradability of lignin debris in the peri-implant area. Clinical trials are needed to confirm the high accuracy in terms of implant positioning of the evaluated surgical guides as well as the intraoral fit and fracture resistance. 

## 5. Conclusions

According to the outcome of this in vitro study, the following conclusions can be drawn:

- Implant placement using FDM and SLA printed surgical guides resulted in comparable accuracy of implant position.
- Both investigated implant positions (SP, SM) showed comparable deviations.
- The use of metal sleeves for surgical guides did not improve the final accuracy of the implant position.

## Figures and Tables

**Table 1:** Mean ± standard deviation of the discrepancies at the implant apex and shoulder level and the main axis.

Position	Technology	Guidance	Apex (mm)			Shoulder (mm)			Axis (°)
			x	y	z	x	y	z	
35	SLA	S	0.3 ± 0.2	−0.2 ± 0.2	−0.1 ± 0.2	0.1 ± 0.1	−0.2 ± 0.2	−0.1 ± 0.1	1.3 ± 0.9
		NS	0.3 ± 0.2	0.1 ± 0.1	−0.1 ± 0.2	0.1 ± 0.1	0.1 ± 0.1	−0.1 ± 0.1	1.6 ± 0.5
	FDM	S	0.2 ± 0.4	−0.3 ± 0.1	0.1 ± 0.3	0.1 ± 0.2	−0.3 ± 0.1	0 ± 0.1	1.9 ± 0.8
		NS	0.3 ± 0.2	−0.1 ± 0.2	0.2 ± 0.3	0.2 ± 0.1	−0.1 ± 0.2	0 ± 0,1	1.9 ± 0.8
37	SLA	S	0.1 ± 0.3	−0.1 ± 0.1	0.0 ± 0.2	0.1 ± 0.1	−0.1 ± 0.1	0.0 ± 0,1	1.2 ± 0.6
		NS	0.1 ± 0.2	0.0 ± 0.1	0.0 ± 0.2	0.1± 0.1	−0.1 ± 0.1	0.0 ± 0,1	0.8 ± 0.5
	FDM	S	0.4 ± 0.4	0.0 ± 0.2	−0.1 ± 0.1	0.3 ± 0.1	0.1 ± 0.2	0.0 ± 0.1	1.7 ± 0.9
		NS	0.3 ± 0.2	0.1 ± 0.1	0.1 ± 0.2	0.2 ± 0.1	0.1 ± 0.1	0.0 ± 0,1	1.3 ± 0.4

SLA: stereolithography, FDM: fused deposition modeling, S: metal sleeve, NS: no metal sleeve, x: sagittal discrepancies, y: transversal discrepancies, z: vertical discrepancies.

**Table 2:** Results of MANOVA with post hoc pairwise comparisons in case of *p* < 0.05.

		MANOVA	Position	Multiple Comparisons		Tukey Test
		Significance		Group (A)	Group (B)	Significance
Apex	x	0.050				
	y	0.001	35	SLA-NS	SLA-S	0.001
					FDM-S	0.001
					FDM-NS	0.045
	z	0.054				
Shoulder	x	0.001	37	SLA-S	FDM-S	0.001
					FDM-NS	0.017
				SLA-NS	FDM-S	0.001
					FDM-NS	0.033
	y	0.001	35	SLA-NS	SLA-S	0.001
					FDM-S	0.001
			37	SLA-S	FDM-NS	0.035
	z	0.352				
Axis		0.033	37	SLA-NS	FDM-S	0.020

MANOVA: multivariate analysis of variance, SLA: stereolithography, FDM: fused deposition modeling, S: metal sleeve, NS: no metal sleeve, x: sagittal discrepancies, y: transversal discrepancies, z: vertical discrepancies.
